# Prevalence and risk factors for intensive care unit acquired weakness: A protocol for a systematic review and meta-analysis

**DOI:** 10.1097/MD.0000000000022013

**Published:** 2020-09-04

**Authors:** Zheng Li, Qian Zhang, Peng Zhang, Ruixiang Sun, Haijiao Jiang, JingJing Wan, Fang Wu, Xiaoye Wang, Xiubin Tao

**Affiliations:** a Graduate College, Wannan Medical College, Wuhu; b School of Nursing, Lanzhou University, Lanzhou; c The First Affiliated Hospital of Wannan Medical College, Wuhu, China.

**Keywords:** intensive care unit-acquired weakness, meta-analysis, prevalence, risk factors, systematic review

## Abstract

**Background:**

Intensive care unit-acquired weakness (ICU-AW) is an acquired neuromuscular lesion and a common occurrence in patients who are critically ill. We will systematically summarize and incorporate the important risk factors and prevalence from previously published multivariate analyses for ICU-AW.

**Methods:**

We will search the PubMed, Embase, Web of Science, and the Cochrane library to identify the relevant studies about the prevalence and risk factors for ICU-AW. Two reviewers will independently review the studies for eligibility according to the inclusion criteria. Two reviewers will independently assess the quality of studies by using the Newcastle–Ottawa scale for nonrandomized studies. Heterogeneity among studies will be estimated by the *I*^2^ statistic.

**Results:**

This systematic review and meta-analysis will provide an evidence of prevalence and risk factors for the ICU-AW.

**Conclusion:**

We hope that our research will contribute to clinicians and public decision making about the ICU-AW.

## 1 Introduction

Intensive care unit-acquired weakness (ICU-AW), defined as “clinically detected weakness in critically ill patients in whom there is no plausible etiology other than critical illness,” is the most common neuromuscular impairment.^[1]^ ICU-AW can be caused by a critical illness polyneuropathy (CIP), a critical illness myopathy (CIM), or muscle disuse atrophy, alone or in combination.^[2]^ ICU-AW is associated with difficulty in weaning from the ventilator, prolonged ICU stay, and higher hospitalization charges and increases long-term morbidity and mortality.^[3,4]^

ICU-AW is detected in 30 to 50% of patients and the incidence is even higher (up to 67%) in critically ill patients with sepsis,^[5]^ and it is still as high as 36% after discharge.^[6]^ Neuromuscular weakness in the ICU is most often due to CIM, CIP, or critical illness neuromyopathy (CINM) a combination of the 2.^[7–9]^

The major histopathologic finding in CIM is relatively selective loss of myosin, which can be identified as a lack of reactivity to myosin ATPase in non-necrotic fibers.^[10,11]^ CIP appears to be a common complication of severe sepsis and is thought to represent a neurologic manifestation of the systemic inflammatory response syndrome.^[12–14]^ There is some correlation with elevations in blood glucose and reductions in serum albumin.^[15,16]^ Sepsis may be a common pathologic mechanism underlying the development of CINM.^[17]^

ICU-AW is diagnosed after the onset of critical illness, weakness is symmetrical and affects all 4 limbs and the respiratory muscles with sparing of the facial muscles.^[18]^ The muscle tone is almost invariably reduced, but deep tendon reflexes can be either reduced or normal.^[19,20]^ The diaphragm is often involved, leading to prolonged mechanical ventilation and difficult weaning.^[21]^

Exact incidence of ICU-AW is unknown because of wide variation in the patient population, risk factors, and the diagnostic criteria used, and in the timing of assessment.^[22]^ There are already many published multivariate analyses on risk factors, including sepsis, multiorgan failure, the systemic inflammatory response syndrome, immobility, duration of vasopressor and catecholamine support,^[23]^ hyperglycemia, renal failure and renal replacement therapy, and so on.^[24–26]^ In this systematic review and meta-analysis, we will summarize and incorporate the important risk factors from previously published multivariate analyses for ICU-AW in critically ill adult patients.

## 2 Methods

### 2.1 Protocol and registration

This systematic review and meta-analysis protocol is based on the preferred reporting items for systematic reviews and meta-analyses statement.^[27]^ This systematic review and meta-analysis has been registered on the International Platform of Registered Systematic Review and Meta-analysis Protocols. The registration number is INPLASY202070080 and the DOI is 10.37766/inplasy2020.7.0080.

### 2.2 Search strategy

We will search the PubMed, Embase, Web of Science, and the Cochrane library from the inception to the August 2020. The search terms are “ICU-AW,” “intensive care unit-acquired weakness,” “CIM,” “critical illness myopathy,” “CIP,” “critical illness polyneuropathy,” “CINM,” “critical illness neuromyopathy,” and “risk factors,” “predisposing factor.” What's more, a manual search of references of relevant review articles will be performed to identify additional studies.

### 2.3 Inclusion and exclusion criteria

In this systematic review and meta-analysis, we will include the studies satisfying the following criteria:

(1) the population are the ICU-AW patients with no restriction on the gender and the age.
(2) The diagnosis of ICU-AW is reliable and have high accuracy such as the Medical Research Council scale or the electrophysiological studies.
(3) the studies reported the prevalence and risk factors of the ICU-AW.
(4) The study design is cross-section or cohort study.

We did not limit the language or the year of publication. We will exclude protocols, editorials, meeting abstracts, and other reviews.

### 2.4 Study selection

EndNote X9 will be used to manage the initial search records. Two reviewers (ZL and QZ) will independently review the titles and abstracts based on the inclusion criteria. We will download the texts of the potential records to review them for inclusion further. Disagreements will be resolved by discussion or through consultation with a third reviewer (XBT). Study selection will be summarized in a Preferred Reporting Items for Systematic Reviews and Meta-Analyses flow diagram.

### 2.5 Data extraction

Two reviewers (ZL and QZ) will extract date independently by each reviewer using a standardized data collection form. We will collect the following date including: the name of the first author, publication year, study location, study design (cross-section study or cohort study), sample size, age, sex, ICU-AW incidence, statistic analysis methods, reported risk factors, and so on. Disagreement will be solved by discussion or by consulting the third person (XBT).

### 2.6 Risk of bias assessment

Two reviewers will independently assess the quality of included studies by using the Newcastle–Ottawa scale for nonrandomized studies.^[29]^ This is a specific method for assessing the quality of cohort and case-control study. The overall quality score ranging from 0 (minimum) to 9 (maximum). Disagreement will be solved by discussion or by consulting the third person (XBT).

### 2.7 Data synthesis

We will use the STATA 15.0 (Stata Corp LP, College Station, TX) to analysis. Odds ratios will be used for quantitative analyses. We will make a forest plot to visually evaluate the odds ratios and corresponding 95% confidence intervals of each risk factor, and use the Chi-square test for hypothesis testing (*P* < .05, considered statistically significant). Sensitivity analysis will be also conducted to assess the impact of a single study on a comprehensive estimate of each risk factor. The degree of heterogeneity will be assessed using the *I*^2^ statistic. *I*^2^ values of 25%, 50%, and 75% indicate low, moderate, and high heterogeneity, respectively. We will use Egger test to evaluate publication bias and small-study effect, and a *P*-value < .1 in the test confirms the bias and small-study effect.^[31]^

### 2.8 Subgroup analysis

We will conduct subgroup analysis to reduce the random variations between the estimates of the primary study. The subgroup analysis will be based on the different quality of studies and the different age and gender of participants.

### 2.9 Quality of evidence of included reviews

We will rate the evidence as “high,” “moderate,” “low,” or “very low” in a conclusive table using the Grading of Recommendations Assessment, Development, and Evaluation system.^[32]^

## 3 Discussion

ICU-AW has high prevalence and always accompany with poor clinical outcomes. It is significant to figure out the incidence and the risk factors of ICU-AW. We hope this systematic review and meta-analysis could provide evidence for the prediction and prevention of ICU-AW.

## Author contributions

Xiubin Tao conceived the idea of research. Zheng Li, Qian Zhang, and Peng Zhang developed the first draft of the manuscript. Peng Zhang, Ruixiang Sun, Haijiao Jiang, JingJing Wan, Fang Wu, and Xiaoye Wang revised several versions of the manuscript.
